# MiR-155 deficiency protects renal tubular epithelial cells from telomeric and genomic DNA damage in cisplatin-induced acute kidney injury

**DOI:** 10.7150/thno.72456

**Published:** 2022-06-06

**Authors:** Qing Yin, Ya-Jie Zhao, Wei-Jie Ni, Tao-Tao Tang, Yao Wang, Jing-Yuan Cao, Di Yin, Yi Wen, Zuo-Lin Li, Yi-Lin Zhang, Wei Jiang, Yue Zhang, Xiao-Yu Lu, Ai-Qing Zhang, Wei-Hua Gan, Lin-Li Lv, Bi-Cheng Liu, Bin Wang

**Affiliations:** 1 Institute of Nephrology, Zhong Da Hospital, Southeast University School of Medicine, Nanjing, Jiangsu, China.; 2 Department of Pediatric Nephrology, the Second Affiliated Hospital of Nanjing Medical University, Nanjing, Jiangsu, China.; 3 Nanjing Medical University, Nanjing, Jiangsu, China.; 4 Institute of Nephrology, Taizhou Clinical Medical School of Nanjing Medical University (Taizhou People's Hospital), Taizhou, Jiangsu, China.; 5 Department of Pediatric Nephrology, Affiliated Maternity and Child Health Care Hospital of Nantong University, Nantong, Jiangsu, China.

**Keywords:** MiR-155, DNA damage, AKI, TRF1, CDK12

## Abstract

**Rationale**: Cisplatin nephrotoxicity is an important cause of acute kidney injury (AKI), limiting cisplatin application in cancer therapy. Growing evidence has suggested that genome instability, telomeric dysfunction, and DNA damage were involved in the tubular epithelial cells (TECs) damage in cisplatin-induced AKI (cAKI). However, the exact mechanism is largely unknown.

**Methods:** We subjected miR-155^-/-^ mice and wild-type controls, as well as HK-2 cells, to cAKI models. We assessed kidney function and injury with standard techniques. The cell apoptosis and DNA damage of TECs were evaluated both *in vivo* and *in vitro*. Telomeres were measured by the fluorescence *in situ* hybridization.

**Results:** The expression level of miR-155 was upregulated in cAKI. Inhibition of miR-155 expression protected cisplatin-induced AKI both *in vivo* and *in vitro*. Compared with wild-type mice, miR-155^-/-^ mice had reduced mortality, improved renal function and pathological damage after cisplatin intervention. Moreover, inhibition of miR-155 expression attenuated TECs apoptosis and DNA damage. These protective effects were caused by increasing expression of telomeric repeat binding factor 1 (TRF1) and cyclin-dependent kinase 12 (CDK12), thereby limiting the telomeric dysfunction and the genomic DNA damage in cAKI.

**Conclusion:** We demonstrated that miR-155 deficiency could significantly attenuate pathological damage and mortality in cAKI through inhibition of TECs apoptosis, genome instability, and telomeric dysfunction, which is possibly regulated by the increasing expression of TRF1 and CDK12. This study will provide a new molecular strategy for the prevention of cAKI.

## Introduction

Acute kidney injury (AKI), characterized by a sharp decline in renal function, remains a significant health burden due to its high morbidity and mortality 1, 2. Cisplatin is one of the major causes of clinical AKI. Recent studies indicated that cisplatin-induced AKI (cAKI) incidence is 20% ~ 30% in patients 3. The reduction of cAKI has been a critical clinical issue that raised great concerns in physicians.

Cisplatin is an important chemotherapy drug for a range of solid tumors, such as testicular cancer, ovarian cancer, small cell lung cancers, and breast cancer 4-6. However, cisplatin therapy is limited by serious side effects such as cAKI, ototoxicity, and neurotoxicity. cAKI mainly occurred in tubular epithelial cells (TECs) 7. Cisplatin covalently binds to DNA and produces a DNA adduct and ultimately leads to DNA damage 8. Previous studies demonstrated that DNA damage caused by cisplatin led to cell apoptosis 9-11. Therefore, an identification of the key molecules regulating DNA damage in TECs may provide a potential therapeutic target for the treatment of cAKI.

MicroRNAs, short non-coding RNAs with a length of 22-24 nucleotides, induce mRNA degradation or block protein translation by binding to the 3'UTR of target mRNA 12. Recent studies found that miRNAs are crucial regulators of cellular function and are promised to yield a new class of therapeutics 13-15. A highly conserved region encodes miR-155 in the third exon of the B cell integration cluster gene, located in human chromosome 21q21 16. Growing evidence has also demonstrated the crucial role of miR-155 in the development of kidney disease. Clinical and preclinical studies have shown that miR-155 expression significantly increases in AKI kidneys 17, 18. In addition, miR-155 is reported to be one of the miRNAs contributing to DNA damage activation 19-21. However, its potential role in the pathogenesis of cAKI and the corresponding DNA damage remains unclear.

Telomeres are TTAGGG repeats that provide binding sites for telomeric-specific protein complexes shelterin, which controls telomeric length and inhibits DNA damage 22. Telomeric repeat binding factor 1 (TRF1) contributes to shelterin formation and protects telomeric integrity by preventing telomeric fusion and fragility 23, 24. TRF1 deficiency leads to telomeric replication errors and DNA damage response (DDR) activation 25, 26. By targeting TRF1, miR-155 increased telomeric fragility and metaphase chromosome structure abnormalities in human breast cancer 27. Cyclin-dependent kinase 12 (CDK12) regulates transcription by affecting serine phosphorylation in RNA polymerase II (RNA pol II CTD) and DDR 28, 29. CDK12 knockout induced the downstream genes silence which causes a “DNA damage repair defect” state, making cells more sensitive to DNA damage 28. Bioinformatic method and literature suggested that TRF1 and CDK12 were potential targets for miR-155 29.

In this study, we posited that miR-155 could initiate renal injury in cAKI through mediated DNA damage. We demonstrated that miR-155 deficiency could significantly attenuate pathological damage and mortality in cAKI through inhibition of TECs apoptosis, genome instability, and telomeric dysfunction, which is possibly regulated by the increasing expression of TRF1 and CDK12. This study will provide a new molecular strategy for the prevention of cAKI.

## Results

### miR-155 deletion ameliorates cisplatin-induced AKI *in vivo*

**Figure 1:**
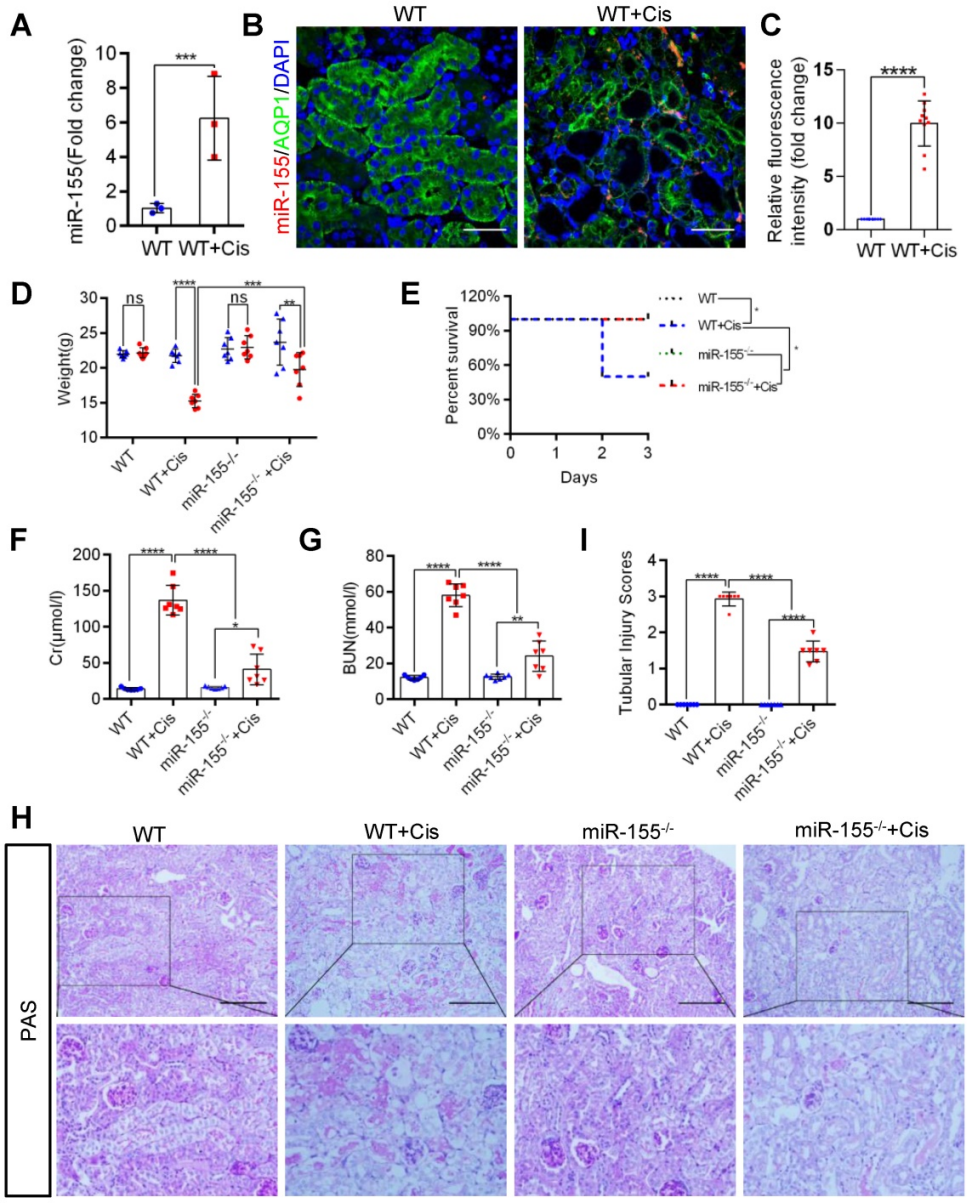
** MiR-155 deletion ameliorates cisplatin-induced AKI *in vivo*. (A)** Real-time PCR analysis of miR-155 levels in wild type and mice kidneys 72 h after cisplatin administration. wild type (n = 3); WT+ Cis (n = 3). **(B and C)** Representative immunofluorescence double staining of AQP1 (green, proximal tubules) and miR-155 (red) in kidneys of mice. Scale bar: 50 µm **(D)** Body weight (0 vs. 72 h), **(E)** Survival after cisplatin injection was monitored until day 3 (n =7). **(F)** Serum creatinine levels and BUN **(G)** over time, 72 h after saline or cisplatin administration (n = 7). **(H and I)** Representative kidney histology as shown by PAS staining 72 h after saline or cisplatin injection. Scale bar: 200 µm. The dot plot showed the corresponding quantification of tubular injury score (n = 7). Data are presented as mean ± SD, * p < 0.05, ** p < 0.01, *** p < 0.001, **** p < 0.0001.

Firstly, we evaluated miR-155 expression in cAKI by qPCR and fluorescence *in situ* hybridization. The miR-155 level significantly increased in the kidney at 72 h after cisplatin treatment (Figure 1A-C). At 72 h of cAKI, wild-type (WT) mice lost 29.8% body weight, while miR-155^-/-^ mice had a 16.6% decline in body weight (Figure 1D). In addition, miR-155 deletion significantly improved the injury in cAKI, leading to decreased mortality, serum creatinine levels, and BUN (Figure 1E-G). Periodic acid-Schiff (PAS) staining and hematoxylin-eosin staining (HE) of kidney sections after cAKI demonstrated mitigation of tubular injury (including TECs swelling, necrosis, cellular debris accumulation, and casts formation) in miR-155^-/-^ kidneys compared with WTs (Figure 1H-I and Figure S1). In conclusion, miR-155 deletion ameliorated cAKI *in vivo*.

### miR-155 inhibition attenuates renal tubular cells apoptosis during cAKI

**Figure 2:**
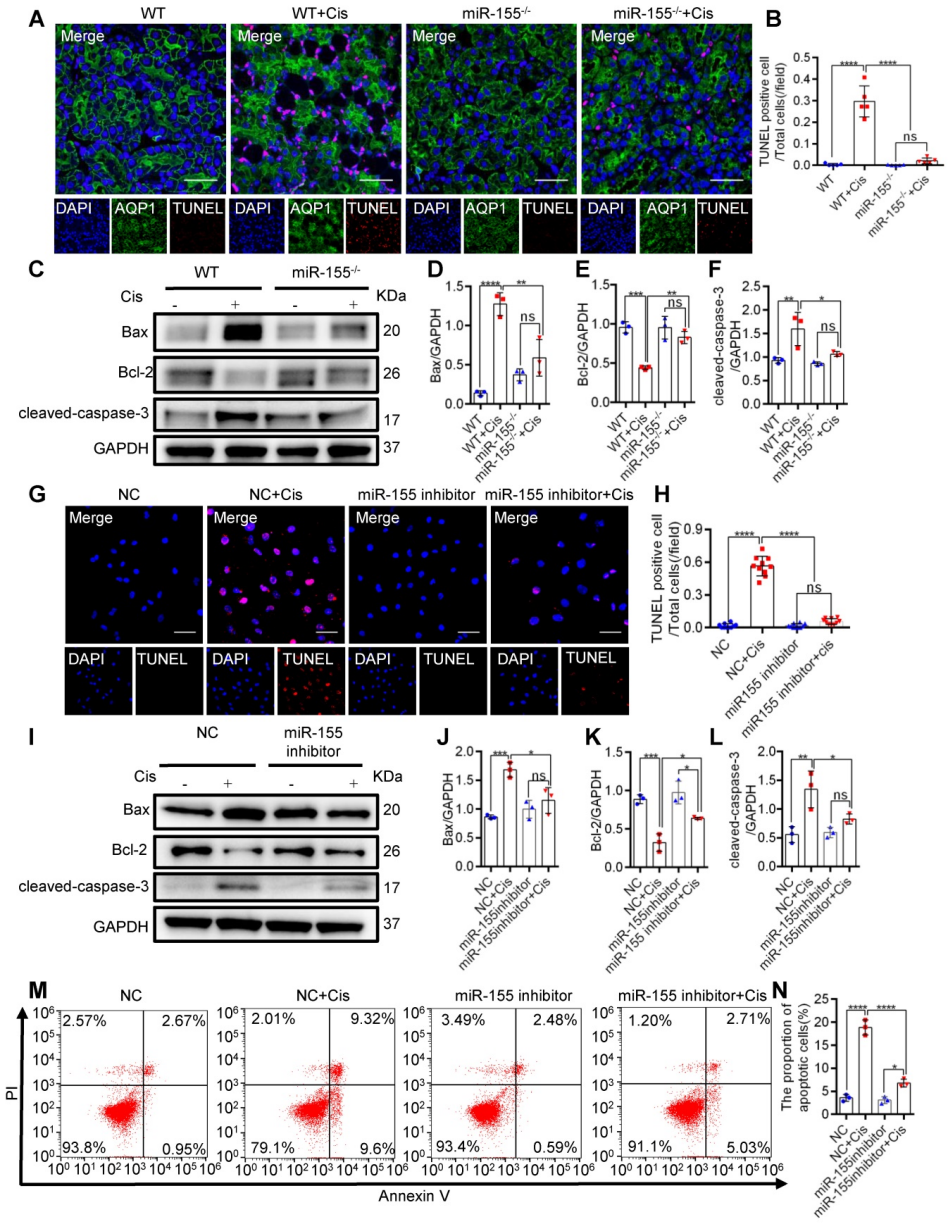
** MiR-155 inhibition attenuates renal tubular cells apoptosis during cAKI. (A)** Representative immunofluorescence double staining of AQP1 (green, proximal tubules) and TUNEL (red) in kidney tissues and quantification of the apoptotic cells (n = 5). Scale bars, 50 µm. **(B)** The quantification of TUNEL^+^ TECs (n = 5). **(C-F)** Representative western blot gel documents and summarized data showing the protein levels of Bax, Bcl-2, and cleaved-caspase-3 in the kidneys of mice with cisplatin-induced AKI. **(G and H)** Representative images of TUNEL staining in HK-2 cells and quantification of the apoptotic cells per field (n = 10). Scale bars, 50 µm. **(I-L)** Representative western blot gel documents and summarized data showing the protein levels of Bax, Bcl-2, and cleaved-caspase-3 in HK-2 cells treated with cisplatin (n = 3). **(M and N)** Flow cytometry analysis of annexin V/PI staining and quantification of the apoptotic cells (n = 3). Data are presented as mean ± SD, * p < 0.05, ** p < 0.01, *** p < 0.001, **** p < 0.0001.

To explore the role of miR-155 in cisplatin-induced apoptosis, we measured terminal deoxynucleotidyl transferase mediated dUTP nick end labeling (TUNEL) staining and apoptosis-related proteins in the kidney of cAKI. TUNEL staining revealed decreased TECs apoptosis in miR-155^-/-^ mice compared with WTs (Figure 2A-B). Western blot analysis of the cAKI kidneys demonstrated that the miR-155^-/-^ mice have decreased Bax and cleaved-caspase-3 and increased Bcl-2 than in WTs (Figure 2C-F). Consistently, miR-155 inhibition markedly reduced the cisplatin-induced HK-2 cells apoptosis *in vitro* (Figure 2G-N). Therefore, our data showed that inhibition of miR-155 effectively protected cisplatin-induced TECs from apoptosis.

### miR-155 inhibition reduces cisplatin-induced DNA damages both *in vivo* and *in vitro*

**Figure 3:**
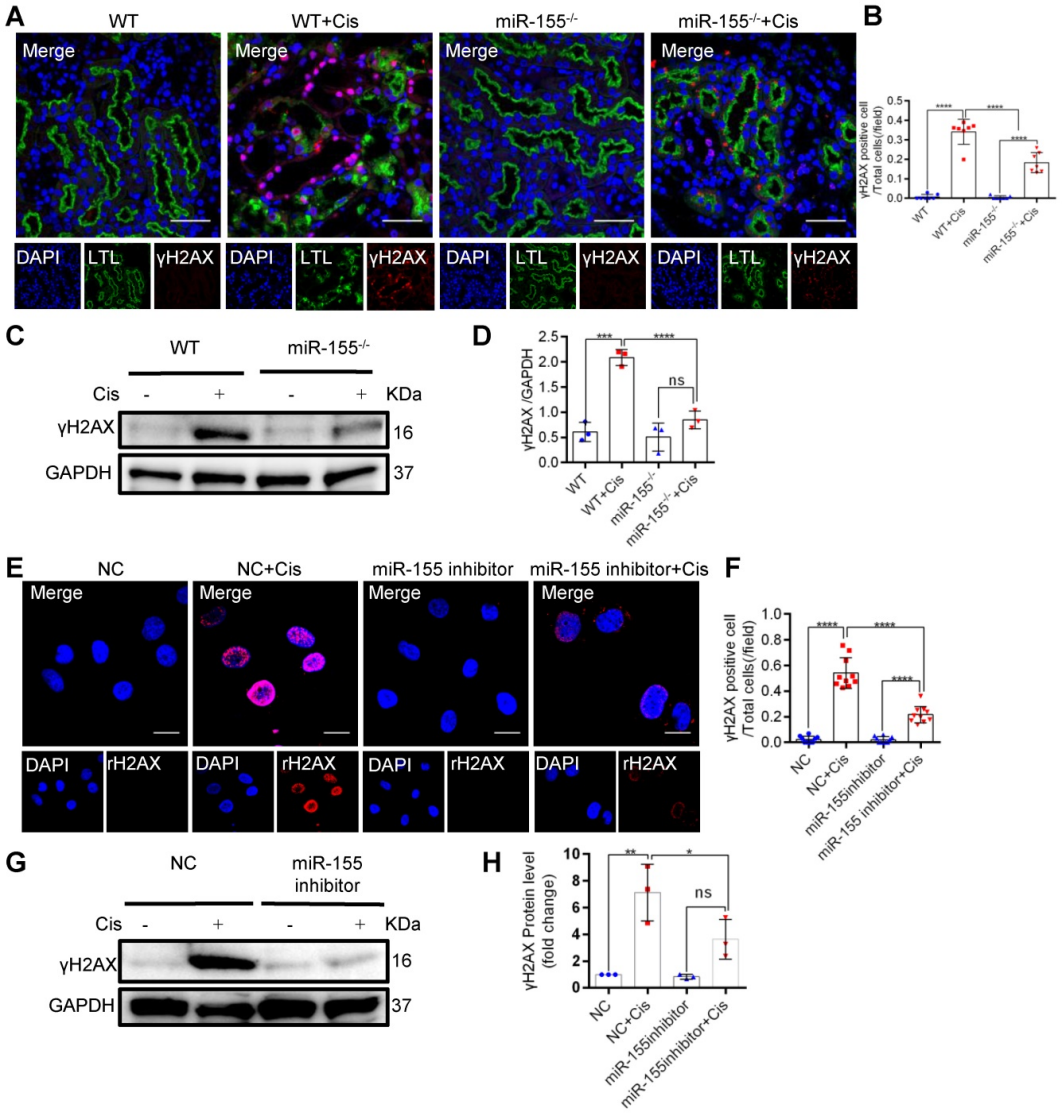
** MiR-155 inhibition reduces cisplatin-induced DNA damages both *in vivo* and *in vitro*. (A)** Representative immunofluorescence double staining of LTL (green, proximal tubules) and γH2AX staining (red) in kidney tissues. Scale bars: 50 µm. **(B)** The quantification of γH2AX^ +^ TECs (n = 7). **(C and D)** Representative western blot gel documents and summarized data showing the protein levels of γH2AX in the kidneys of mice with cisplatin-induced AKI. **(E and F)** Representative images of γH2AX staining in HK-2 cells and quantification of the γH2AX positive cells per field (n = 10). Scale bars, 20 µm. **(G and H)** Representative western blot gel documents and summarized data showing the protein levels of γH2AX in HK-2 cells treated with cisplatin (n = 3). Data are presented as mean ± SD, * p < 0.05, ** p < 0.01, *** p < 0.001, **** p < 0.0001.

In mammalian cells, histone H2AX with phosphorylated Ser139, γH2AX, accumulates at the DNA damage site and is accepted as a DNA damage marker 30. At 72 h after cisplatin intervention, γH2AX expression significantly increased in kidneys of WT but less in miR-155^-/-^ mice (Figures 3A-D). Furthermore, miR-155-inhibition abrogated γH2AX expression in cisplatin-treated HK-2 cells (Figure 3E-H). These data suggested that miR-155 inhibition protected cisplatin-induced DNA damage both *in vivo* and *in vitro*.

### miR-155 inhibition protects cisplatin-induced telomeric dysfunction in renal epithelial cells

**Figure 4:**
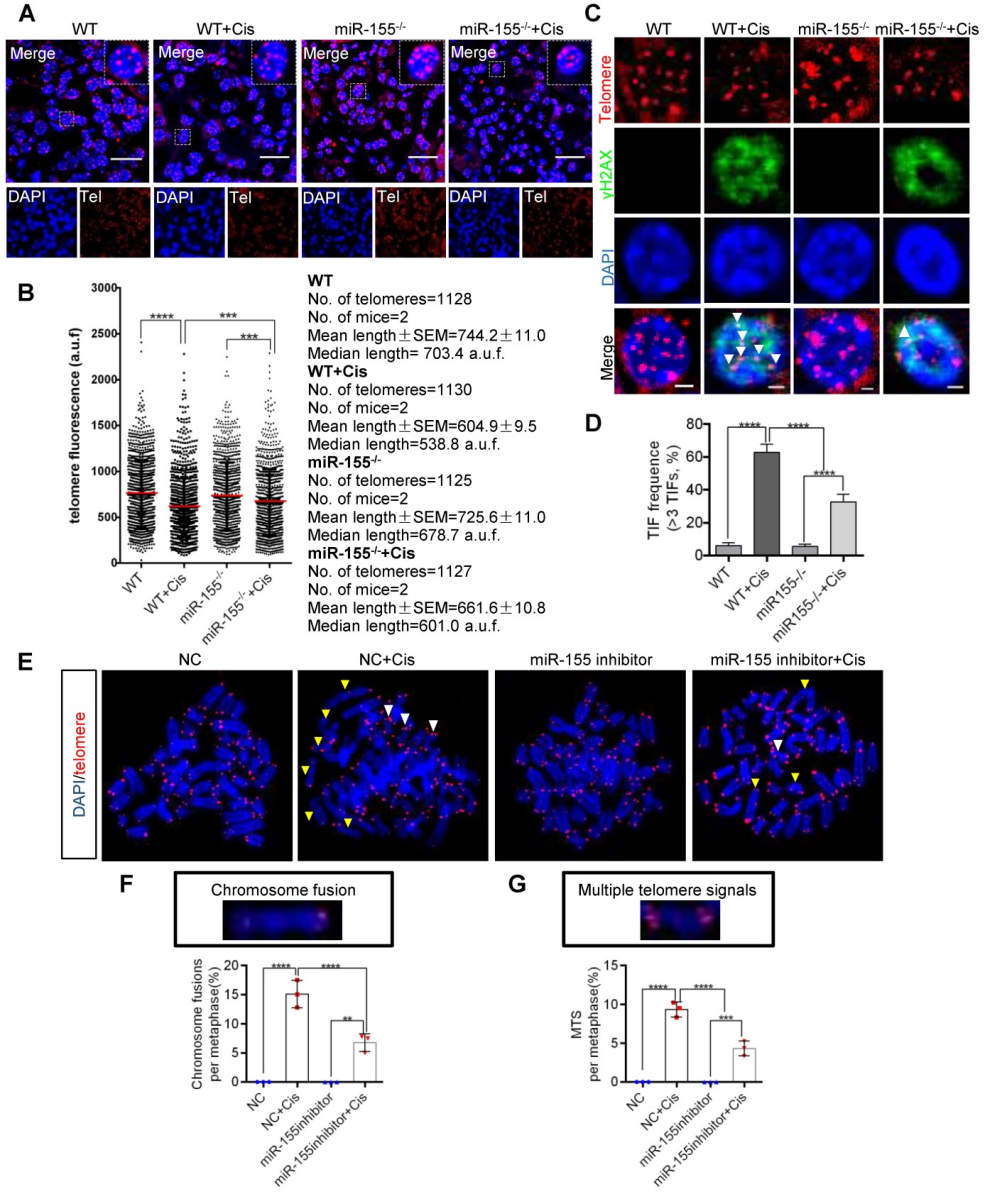
** MiR-155 inhibition protects cisplatin-induced telomeric dysfunction in renal epithelial cells. (A)** Representative images of telomere-stained kidney sections from wild type and miR-155^-/-^ mice 72 h after saline or cisplatin injection. Scale bars: 20 µm. **(B)** Telomeric length measurements by quantitative telomeric DNA FISH in the kidney sections; a.f.u, arbitrary fluorescence units. **(C and D)** Telomeric DNA damage was detected by the co-localization of γH2AX (green) and telomeres (red) in the immunofluorescence and quantification of the percentage of cells with γ-H2AX-positive TIFs in the renal tubular cells of each group of mice. N = 3, n (WT) = 46; n (WT + Cis) = 45; n (miR-155^-/-^) = 40; n (miR-155^-/-^ + Cis) = 43; N, number of independent experiments; n, number of analyzed nuclei. Scale bars: 2 µm. **(E)** Representative images of metaphases from the cells treated with cisplatin *in vitro*. Yellow arrows indicate chromosome fusion. White arrows indicate MTS. Scale bars: 10 µm. **(F and G)** Quantification of the number of chromosome fusions and MTS in TEC metaphases. N = 3, n (WT) = 806; n (WT + Cis) = 796; n (miR-155^-/-^) = 810; n (miR-155^-/-^ + Cis) = 798; N, number of independent experiments; n, number of analyzed chromosomes. TIFs, telomeric dysfunction induced foci, MTS, multiple telomeric signals.* p < 0.05, ** p < 0.01, *** p < 0.001, **** p < 0.0001.

Telomeres locate at the ends of chromosomes, which maintain gene stability by preventing chromosome degradation, fusion, and recombination 31, 32. As knockout of miR-155 attenuates DNA damage, we wondered whether miR-155 influences telomeres in renal epithelial cells during cAKI. This study measured telomeric length by the quantitative FISH (Q-FISH) method. The telomere fluorescence signal intensity significantly decreased in WT mice but less decrease in miR-155^-/-^ mice after cisplatin treatment (Figure 4A-B). Previous studies have shown that γH2AX was associated with critically short/dysfunctional telomeres, also known as telomeric dysfunction-induced foci (TIFs) 33. By performing γH2AX immunofluorescence and telomere DNA FISH, we detected 62.80% of lesions carrying >3 TIFs in kidney sections from cisplatin-injected WTs, while 32.86% of lesions in miR-155^-/-^ kidneys carrying >3 TIFs after cisplatin treatment (Figure 4C-D).

Multiple telomere signals (MTSs) indicate fragile telomeres, arising from replication fork stalling on repetitive telomere sequences. Fragility causes chromosome breaks and genome instability 23. Compared with negative control (NC), application of miR-155 inhibitor inhibited cisplatin-induced MTS and chromosome fusion (Figure 4E-G) and TIFs (Figure S2) *in vitro*. Therefore, miR-155 contributed to telomeric DNA damage after cisplatin stimulation and miR-155 inhibition protected cisplatin-induced telomeric function in renal epithelial cells.

### miR-155 deletion ameliorates cisplatin-induced telomeric DNA damage by enhancing TRF1

**Figure 5:**
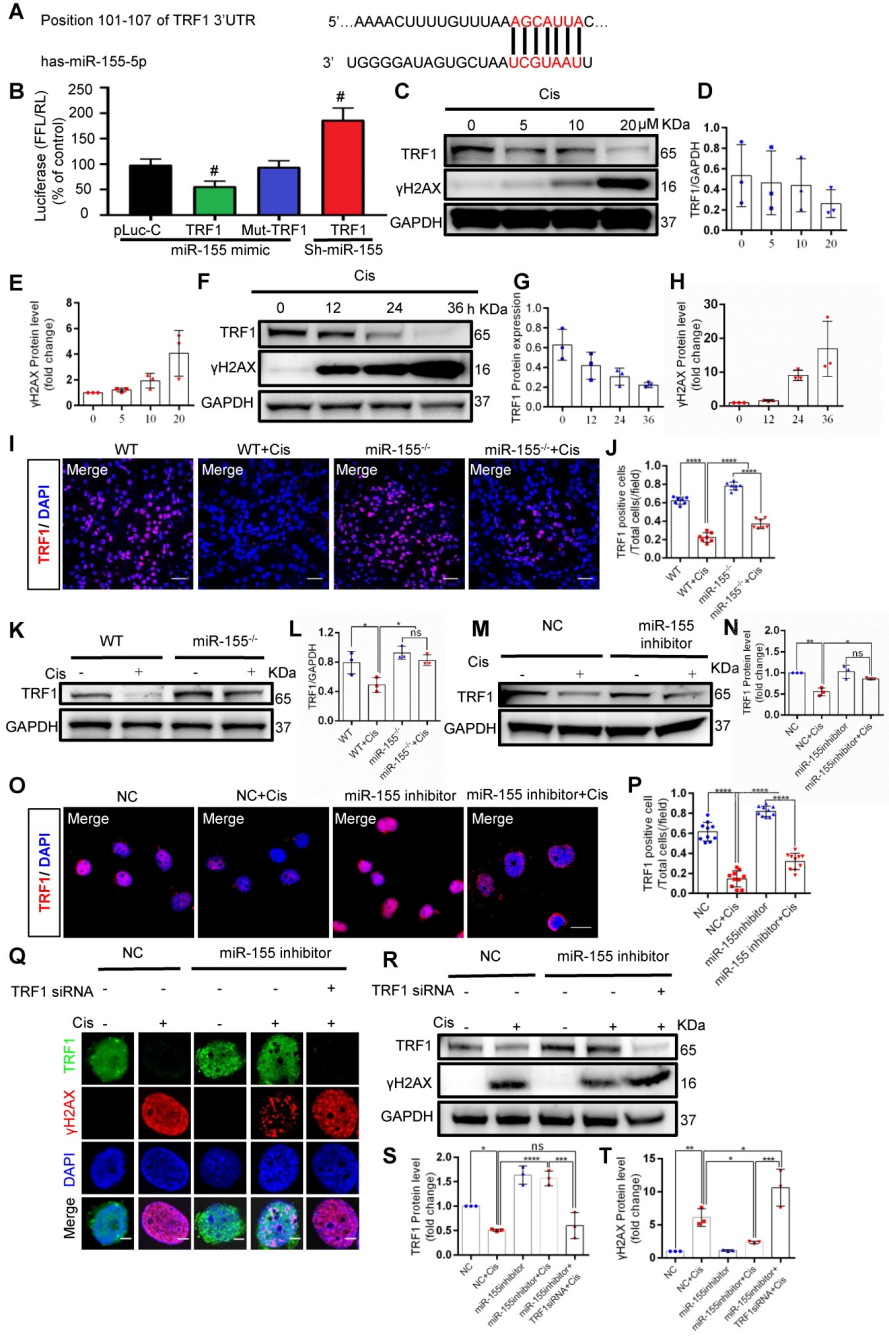
** MiR-155 deletion ameliorates cisplatin-induced telomeric DNA damage by enhancing TRF1. (A)** The schematic diagram depicted the predicted binding site of has-miR-155 targeting the 3'-UTR of TRF1. **(B)** Luciferase reporter assay determined TRF1 as a bona fide target of miR-155 (n = 3). **(C-E)** Western blotting of γH2AX and TRF1 in HK-2 cells (n = 3). HK-2 cells were treated with cisplatin 0, 5, 10, 20 µM for 24 h. **(F-H)** Western blotting of γH2AX and TRF1 in HK-2 cells (n = 3). HK-2 cells were treated with cisplatin 20 µM for 0, 12, 24, 36 h. **(I and J)** Representative images of TRF1-stained kidney sections from wild type and miR-155^-/-^ mice 72 h after saline or cisplatin injection(n = 7). Scale bars: 50 µm. **(K and L)** Western blotting of TRF1 in kidney tissues from wild type and miR-155^-/-^ mice 72 h after saline or cisplatin injection (n = 3). **(M and N)** Western blotting of TRF1 in HK-2 cells (n =3). **(O and P)** Representative images of TRF1-stained in HK-2 cells treated with cisplatin or saline for 24 h (n = 10). Scale bars: 20 µm. **(Q)** Representative images of γH2AX-and TRF1-stained sections of HK-2 cells. Scale bars: 2 µm. **(R-T)** Western blotting of γH2AX and TRF1 in HK-2 cells (n = 3). Data are presented as mean ± SD, * p < 0.05, ** p < 0.01, *** p < 0.001, **** p < 0.0001.

Previous studies have demonstrated that TRF1 deletion causes telomeric DNA damage 23, 26. A conserved binding site of miR-155 was predicted in the TRF1 3'-UTR (site: 101-107) (Figure 5A). We then performed a dual luciferase reporter assay, and the results showed that the activity of luciferase reporters was markedly reduced by miR-155 mimic, while the activity of the TRF1 3'UTR-mutant luciferase reporter was not affected. In addition, miR-155 inhibition significantly increased luciferase activity in TRF1 3'UTR transfected cells (Figure 5B). TRF1 level was decreased in a time- and dose-dependent manner after cisplatin treatment (Figure 5C-H), while miR-155 was elevated (Figure S3). The cisplatin-induced TRF1 reduction was significantly restored in miR-155^-/-^ mice compared with WTs (Figure 5I-L). Consistently, miR-155 inhibitor stimulated TRF1 expression *in vitro* (Figure 5M-P). The miR-155 inhibitor inhibited cisplatin-induced γH2AX expression in HK-2 cells. Moreover, the siRNA-induced TRF1 knock-down blocked protective effects of miR-155 inhibitor in DNA damage after cisplatin stimulation (Figure 5Q-T). Thus, these data demonstrated that miR-155 inhibition rescued telomeric DNA damage by enhancing TRF1.

### miR-155 deletion alleviates cisplatin-induced genomic DNA damages by enhancing CDK12 levels

**Figure 6:**
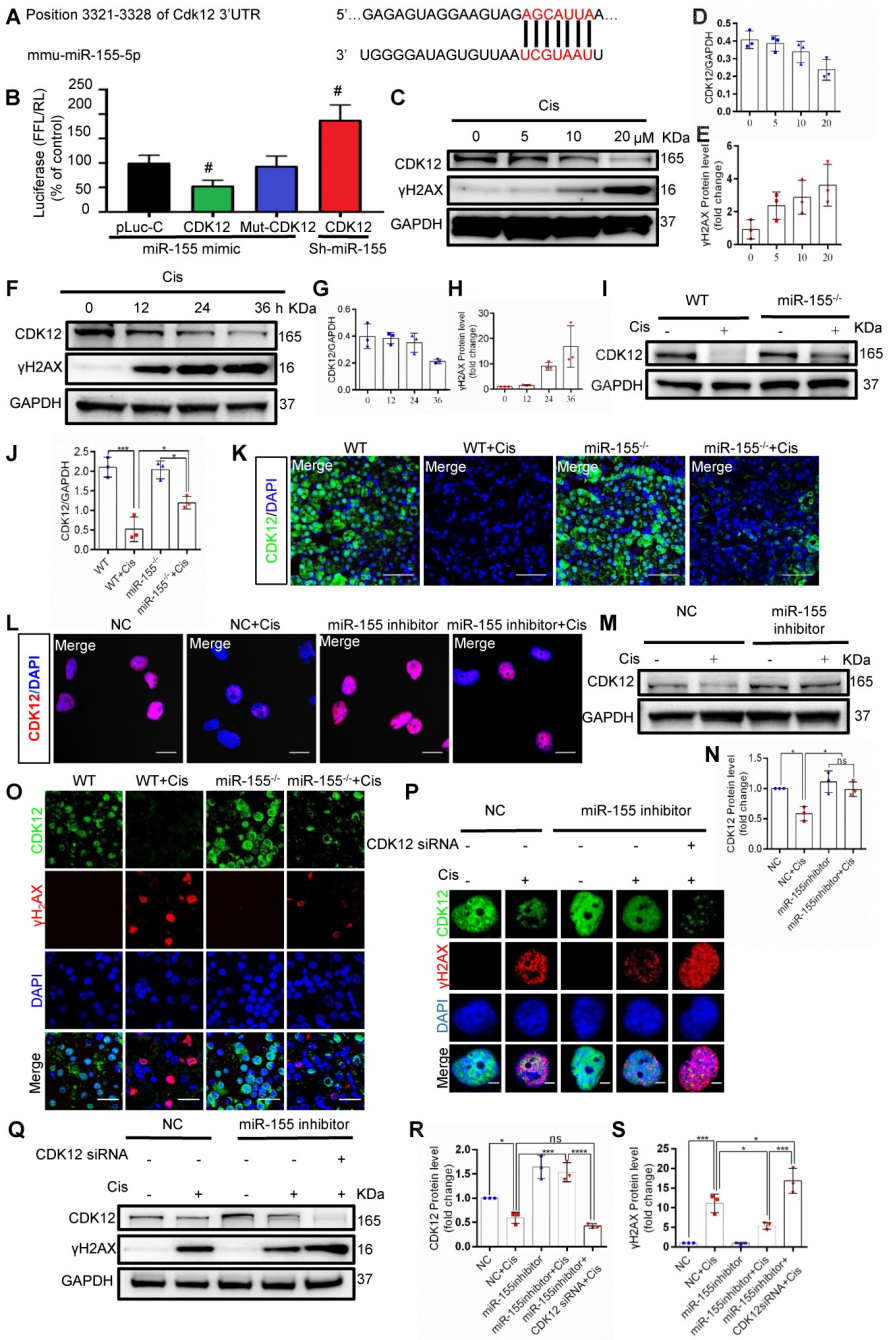
** MiR-155 deletion alleviates cisplatin-induced DNA damages by enhancing CDK12 levels. (A)** The schematic diagram depicted the predicted binding site of miR-155 targeting the 3'-UTR of CDK12. **(B)** Luciferase reporter assay determined CDK12 as a bona fide target of miR-155 (n = 3). **(C-E)** Western blotting of γH2AX and CDK12 in HK-2 cells (n = 3). HK-2 cells were treated with cisplatin 0, 5, 10, 20 µM for 24 h. **(F-H)** Western blotting of γH2AX and CDK12 in HK-2 cells (n = 3). HK-2 cells were treated with cisplatin 20 µM for 0, 12, 24, 36 h. **(I and J)** Western blotting of CDK12 in kidney tissues from wild type and miR-155^-/-^ mice 72 h after saline or cisplatin injection (n = 3). **(K)** Representative images of CDK12-stained kidney sections from wild type and miR-155^-/-^ mice 72 h after saline or cisplatin injection. Scale bars: 50 µm. **(L)** Representative images of CDK12-stained in HK-2 cells treated with cisplatin or saline for 24 h. Scale bars: 20 µm. **(M and N)** Western blotting of CDK12 in HK-2 cells (n = 3). **(O)** Representative images of γH2AX -and CDK12-stained sections of kidney sections from wild type and miR-155^-/-^ mice 72 h after saline or cisplatin injection. Scale bars: 20 µm. **(P)** Representative images of γH2AX-and CDK12-stained sections of HK-2 cells. Scale bars: 2 µm. **(Q -S)** Western blotting of γH2AX and CDK12 in HK-2 cells (n = 3). Data are presented as mean ± SD, * p < 0.05, ** p < 0.01, *** p < 0.001.

Beyond telomeric DNA damage, we observed increased γH2AX signals in the TECs nuclei both *in vivo* and *in vitro*, which were reversed by miR-155 inhibition. Therefore, we further explored the potential mechanism that miR-155 inhibition alleviating cisplatin-induced genomic DNA damages. CDK12 is a transcription-related cyclin kinase and maintains genome stability 28, 34. TargetScan predicts a conserved binding site of miR-155 in CDK12 3'-UTR (Figure 6A). A luciferase reporter was employed for detection and the results demonstrated that the activity of luciferase reporters was clearly reduced by miR-155 mimic. Furthermore, the activity of CDK12-3'-UTR-mutant luciferase reporter was not affected by the miR-155 mimic, while miR-155 inhibition significantly increased luciferase activity in CDK12 3'UTR transfected cells, suggesting that CDK12 was the direct target gene of miR-155 (Figure 6B). In addition, CDK12 decreased in a time- and dose-dependent manner as γH2AX increased after cisplatin treatment (Figure 6C-H), while miR-155 was elevated (Figure S3). In Figure 6I-6K, CDK12 levels were higher in miR-155^-/-^ mice than WTs at 72 h after cisplatin stimulation. Similarly, miR-155 inhibition enhanced CDK12 expression in cisplatin-treated HK-2 cells (Figure 6L-N).

Cisplatin stimulation increased the number of γH2AX-positive cells and inhibited the expression of CDK12 in WTs' kidneys, which was blocked by miR-155 deficiency (Figure 6O). The results obtained from the miR-155 inhibition *in vitro* are consistent with those *in vivo* (Figure 6P-S). Knocked down of CDK12 and miR-155 aggravated DNA damage (Figure 6P-S). In conclusion, these data indicated that miR-155 deletion ameliorated cisplatin-induced genomic DNA damages in renal tubular cells by increasing CDK12 expression.

## Discussion

**Figure 7:**
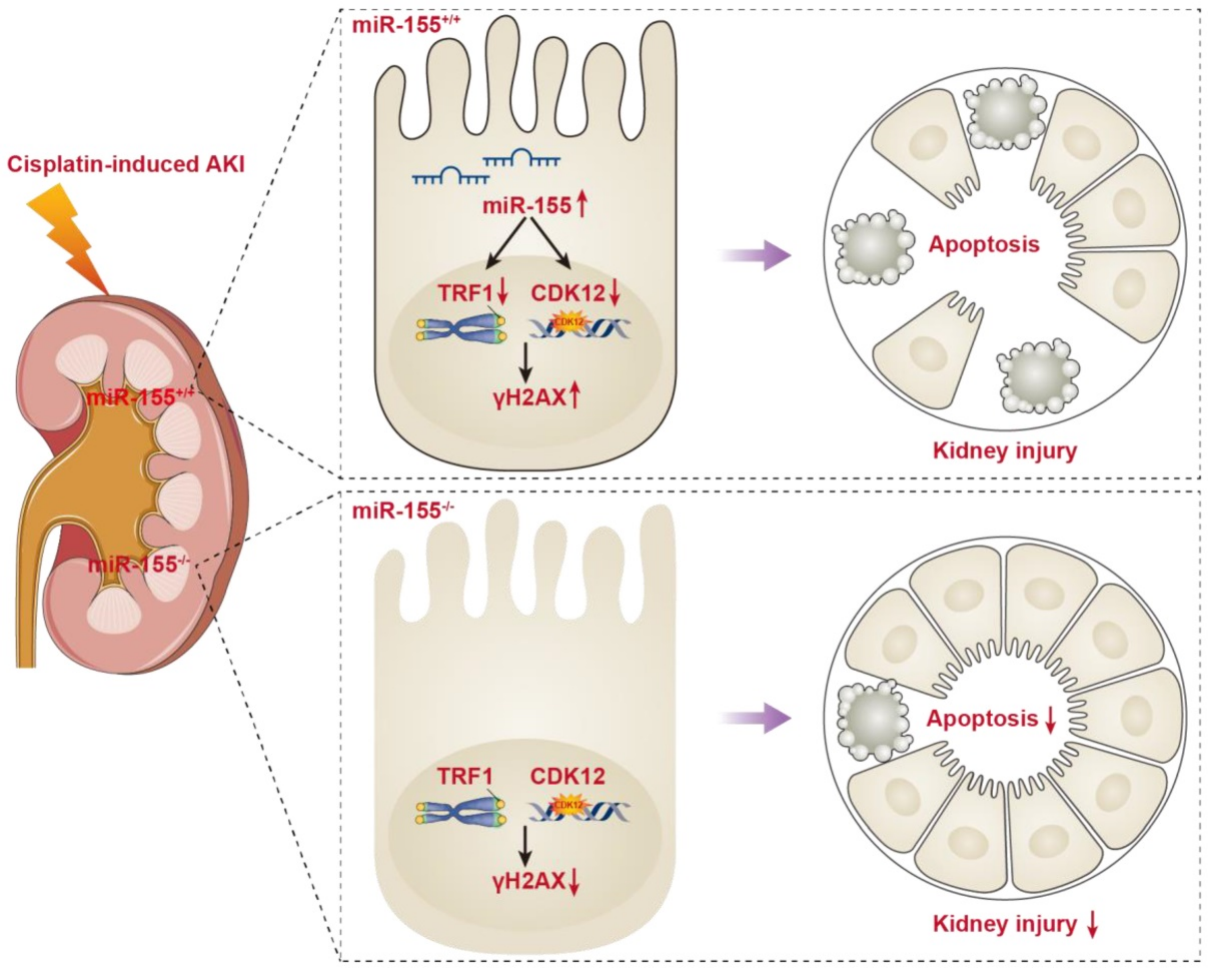
** Schematic illustration of the mechanism of miR-155 inhibition and improvement of AKI induced by cisplatin.** DNA damage in TECs led to apoptosis in cisplatin-induced AKI. MiR-155 deletion significantly protected cisplatin-induced renal injury and reduced mortality in AKI mice. It not only stabilized telomeres by increasing TRF1 expression but also reduced DNA damage by upregulating CDK12. In general, miR-155 inhibition increased genomic stability and reduced DNA damage in cAKI.

In the present study, miR-155 knockout mice presented with the improvement of renal damage and mortality, accompanied by alleviated telomeric DNA damage, extenuated telomeric fragility and chromosome fusion, and abrogated genomic DNA damage in cAKI. Of note, both TRF1 and CDK12 were identified as the targets for miR-155. More importantly, miR-155 deficiency protects TECs from telomeric and genomic DNA damage via increasing TRF1 and CDK12 expression respectively in cAKI. Hence, our findings confirmed the core role of miR-155/TRF1 and miR-155/CDK12 axis in cisplatin-induced TECs injury, and may provide a new molecular strategy to treat cAKI (Figure 7).

As we know, nephrotoxicity or cAKI, is still a big challenge to limit the clinical application of cisplatin 35. Accumulation evidence suggested that inhibition of DNA damage would prevent TECs injury and thus mitigate the cAKI 36, 37. MiR-155, an inflammatory associated miRNA, was also involved in the regulation of DNA damage 19, 20. Previous studies have found that miR-155 can degrade or inhibit protein translation by specifically binding to the 3'UTR regions of mRNAs of multiple different target genes, participating in a variety of cellular biological processes 38. In diabetic nephropathy, it was found that miR-155-5p promoted autophagy and attenuated interstitial fibrosis by targeting PTEN 39. In addition, miR-155-5p promoted renal fibrosis in both diabetic nephropathy and UUO mouse models by inhibiting the sirt1-regulated autophagy pathway 40, 41. Moreover, inhibition of miR-155 was found to be protective from TECs injury in ischemia/reperfusion (I/R) and gentamicin-induced AKI models 17. However, these findings were not enough to prove a pathogenic relevance of miR-155 in cAKI. Some studies have suggested that miR-155 inhibition might promote cell apoptosis 42, 43. In the present study, we impressively demonstrated that miR-155 knockout played a renal protective role in cAKI by blocking the apoptotic response to DNA damage (Figure 1-3). There are many rational explanations for this dual effect. Firstly, miR-155 may have different targets in different diseases 40, 41. Secondly, miR-155 may variably target opposite factors within the same cells as reported for miR-196b 44. Accordingly, the effects of miR-155 in diseases depend on various factors, such as the severity and types of cell damage, and cellular context. In addition, miR-155 is an inflammatory associated miRNA, and inflammation is an important aspect of cisplatin-induced renal injury 45, 46. Whether miR-155 plays a renal protective role by reducing renal inflammation needs to be further studied.

Telomeres are special structures at the ends of chromosomes, which protect the chromosome end from being recognized as DNA double strand breaks 32. Telomeric shortening causes chromosome instability, cellular senescence, and apoptosis 33, 41. The interplay between telomeric shortening and kidney injury is not completely unraveled yet 47, 48. Compared with WT mice, mice with shortened telomeres suffered from I/R injury with greater impairment of renal function and increased acute and chronic histopathological damage. Beyond that, these mice also showed a significant reduction in long-term regenerative capacity of kidney 47. Of interest, our study firstly found miR-155 inhibition improved the TECs telomeric dysfunction in cAKI, as shown by reduced telomeric DNA damage and less telomeric shortening (Figure 4). Hence, maintaining normal telomeric function is one of the key mechanisms by which miR-155 knockout plays a protective role in cAKI.

Telomeres and shelterin protect telomeres from abnormal damage signals and DDR, and maintain the stability of chromosome structure 49. Loss of any shelterin proteins can lead to telomeric instability. Studies have proved that TRF1, an important part of shelterin, was essential in telomeric DDR and telomeric stability 23, 43. In human breast cancer specimens, miR-155 was up-regulated and drove telomeric fragility by negatively regulating TRF1 expression. However, the authors verified that miR-155 could only target TRF1 3'UTR in human species rather than in mouse 27. Interestingly, we found that miR-155 could negatively regulate the expression of TRF1 both in human and mouse. Of note, the targeted TRF1 3'UTR area of miR-155 was indeed different between human and mouse (Figure 5A-B and Figure S4). Collectively, our data clarified the pathogenic role of miR-155 in cAKI and may explain why the renal telomeric dysfunction of miR-155^-/-^ mice was alleviated when cAKI occurred.

Chromosomal DNA consists of genomic DNA (self-replicating DNA), centromeric DNA and telomeric DNA, which ensure the genetic stability of a cell by replicating properly. Beyond telomeric DNA damage, we observed other DNA damage signals in the TECs nuclei, which were also reversed by miR-155 inhibition (Figure 4). Since miRNAs are able to regulate multiple genes simultaneously, it is therefore possible that other genes could also be regulated by miR-155 during cAKI. CDK12 is a member of the CDK family, which phosphorylates RNA polymerase Pol II and promotes transcription expansion 34. In a breast cancer study, CDK12 was proved to be associated with DNA damage 50. Spontaneous DNA damage occurred in the primary cells obtained from CDK12^-/-^ embryos 28. This study firstly identified that CDK12 as a novel target of miR-155 in cAKI. while miR-155 loss enhanced CDK12 expression, thereby reducing genomic DNA damage in cAKI. These findings shed a new light on the important role of miR-155/CDK12 axis in cAKI development.

## Conclusions

In this study, miR-155 inhibition was found to play a protective role in kidney injury by compacting nuclear chromatin to inhibit the DNA damage-apoptosis pathway. More importantly, we firstly demonstrated that inhibition of miR-155 killed two birds with one stone, as indicated by improved telomeric DNA damage via restoring TRF1 expression and limited genomic DNA damage via enhancing CDK12 expression. Therefore, targeted inhibition of miR-155 in TECs is an important strategy for the prevention and treatment of cAKI.

## Materials and Methods

### Mice

MiR-155^-/-^ mice in a C57BL/6 background were provided by Dr. Jie Du (Beijing Anzhen Hospital, Capital Medical University, Beijing, China), which were originally purchased from The Jackson Laboratory (Bar Harbor, ME), and at 8-10 weeks old with 21-24 g body weight. C57BL/6 (wild-type) mice were aged and weight-matched (Vital River Laboratory Animal Technology Co., Ltd.). Experimental procedures were approved by the ethics committees for animal experimentation of Southeast University (No. 20191101004). AKI was induced by a single intraperitoneal injection of cisplatin (18 mg/kg, Sigma), while control groups were injected with saline only. Mice were sacrificed after 3 days, and kidney tissue was collected for various studies. Blood samples are taken before the sacrifice and transferred into eppendorf tubes. The plasma was separated by centrifugation at 3000 rpm at 4 °C for 30 min, which was collected for further analysis. Serum creatinine (SCr) was detected by a creatinine assay kit (Jiancheng). Blood urea nitrogen (BUN) was detected by an assay kit (Jiancheng).

### Morphological studies and tubular injury scoring

In brief, formalin-fixed, paraffin-embedded kidney sections (4-μm thick) stained with hematoxylin and eosin (HE) and periodic acid-Schiff (PAS) staining were used to assess kidney morphology. A semi-quantitative score of renal tubular injury was performed according to the following methods: 0, no injury; 1, <25%; 2, 25~50%; 3, 50~75%; 4, >75%. The average score of 10 random sections was calculated as the tubular injury score.

### Cell culture, transfection, and Cell treatment

Human renal tubular epithelial cell line HK-2 (American Type Culture Collection) and mouse tubular epithelial cells (mTECs; a gift from J. B. Kopp, National Institutes of Health) were cultured in DMEM/F12 supplemented with 10% FBS in a 37 °C incubator with 5% CO_2._ MiR-155 inhibitors, TRF1 siRNA, CDK12 siRNA, and negative control (NC) were designed and synthesized by Hanbio (Shanghai, China). The sequences of miR-155 inhibitors and corresponding controls were 5'-AACCCCUAUCACGAUUAGCAUUAA-3' and 5'- UCUACUCUUUCUAGGAGGUUGUGA-3', respectively. The sequences of TRF1 siRNA, CDK12 siRNA, and NC were 5'-CCAAAUUCUCAUAUGCCUUTT-3', 5'- GCCAGCAUUUAGUCAGGUUTT-3' and 5 '-UUCUCCGAACGUGUCACGUTT-3, respectively. HK-2 cells were transfected with Lipofectamine 3000 (Invitrogen) according to the manufacturer's protocol. In short, cells were seeded in 6-well plates (2-3×10^5^ cells/well) and cultured to 60-80% confluence. The transfection complex was prepared according to the manufacturer's instructions and added directly to the cells. The final concentration of TRF1 siRNA, CDK12 siRNA, miR-155 inhibitor, and NC was 100 nM. Then 20 uM of cisplatin was added after transfection.

### Western blotting

A 100× protease inhibitor was added with RIPA lysis buffer (Servicebio) to extract total proteins from kidney and transfected HK-2 cells, and SDS-PAGE isolated 4% to 20%. The proteins were then transferred to PVDF membranes (Millipore) and were blocked in NcmBlot blocking buffer (NCM Biotech) at room temperature for 10 min. Then membranes were incubated overnight with primary antibodies as follows: anti-TRF1 (ab1423, Abcam), anti-γH2AX (ab26350 Abcam), anti-CDK12 (GTX130809, GeneTex), anti-Bcl-2 (3498, Cell Signaling Technology), anti-Bax (89477, Cell Signaling Technology) and anti-cleaved-caspase-3 (9661s, Cell Signaling Technology). Secondary antibodies were used for detection by an ECL advanced system (GE Healthcare). Intensity values expressed as the relative protein expression were normalized to GAPDH (ab2000, Abways). The gray bands were analyzed with ImageJ software (NIH, Bethesda, MD, USA) to compare the expression between targeted proteins and internal controls.

### Detection of mRNA and miRNA

RNAiso (Takara) extracted total RNA from the kidney tissue and HK-2 cells. After that, we collected 10 ng RNA for further analysis. Mature miRNAs were quantified using miRNA-specific primers (GeneCopoeia) real-time PCR assay kit. U6 was selected as internal standards for gene expression, respectively. Relative gene expression was calculated using the 2^-ΔΔCT^ formula.

### Fluorescence *in situ* hybridization (FISH)

Proteinase K (20 μg/mL) was used to digest 4 μm paraffin kidney tissue sections (deparaffinized and hydrated). It was then prehybridized in pre-hybridization buffer for 8 min (78 °C), followed by hybridization using Cy3-labeled miR-155 probes (devised by Genepharma, Shanghai, China) for 5 min (73 °C) and overnight at 37 °C. The sections were then washed with saline-sodium citrate buffer at 43 °C to remove the unhybridized probes. The images of FISH were taken under a confocal microscope.

### TUNEL Staining Assay

The whole kidney was fixed with 10% formaldehyde for 24 h, embedded in paraffin and sected (thickness 4 μm). 4 μm thick renal tissue sections were stained with a TUNEL staining kit (Beyotime) according to the manufacturer's instructions.

### Immunofluorescence staining

Immunofluorescence analysis was performed on 4 μm thick renal tissue sections and HK-2 cells. They were performed with anti-TRF1 (ab1423, Abcam), anti-γH2AX (ab26350), anti-CDK12 (ab246887, Abcam) , AQP1 (ab 9566, Abcam) , LTL (FL-1321-2, Vector Laboratories) and incubated with secondary antibodies (ab150114 and ab150077, Abcam). Under the confocal microscope, 10 fields of view were randomly assigned, and the number of positive tubules and positive cells was counted in a blind manner.

### Flow cytometry analysis of cell apoptosis

Flow cytometry analysis of cell apoptosis was performed using the Annexin V-FITC apoptosis detection kit (KGA108, KeyGEN BioTECH). Briefly, HK-2 cells were detached by trypsinization and harvested by centrifugation at 2,000 rpm for 5 min. Then, the cells were washed twice with PBS. The cells were resuspended in a binding buffer at a density of 1-5×10^6^ cells/mL. The single cell suspension of 500 μL was incubated with 5 μL of Annexin V-fluorescein isothiocyanate and 5 μL of propidium iodide for 15 min at room temperature. Finally, the mixture was analyzed with a flow cytometer.

### Dual-luciferase Reporter Assay

HK-2 cells and mTECs were co-transfected with 3'-UTR-specific luciferase reporter constructs (3'UTR-NC, 3'UTR-TRF1, 3'UTR-TRF1-mutant, 3'UTR-CDK12, 3'UTR-CDK12-mutant), miRNA (miRNA-NC or miR-155-5p), and Renilla luciferase using lipofectamine 3000 (Invitrogen). After 48 h of transfection, the luciferase activity of cells was measured using a Dual Luciferase Assay Kit (Promega, E1910) and microplate reader (Tecan M1000). Luciferase activity of each groups was calculated and graphed. Renilla luciferase was used to normalize the value of firefly luciferase.

### Telomere Q-FISH Analysis

Quantitative telomere fluorescence *in situ* hybridization (Q-FISH) directly on kidney sections was performed as previously described 51. Telomere software was used to quantify the fluorescence intensity of telomeres. Two different researchers carried out the analysis in a blind way and 10 random images were analyzed. Results were expressed in telomere fluorescence intensity.

Telomeric dysfunction-induced lesions (TIFs) — To determine the presence of telomeric DNA damage signals, the telomeres were labeled with Cy3-labeled PNA telomere probes (Panagene F1002), followed by immunofluorescence staining for γH2AX. γH2AX immunostaining was performed with γH2AX antibody (ab26350, Abcam) overnight, followed by a secondary antibody. TIFs were quantitated manually by measuring the co-location of telomere probes and γH2AX lesions.

### Statistical analysis

Data was expressed as mean ± standard deviation (SD). Comparison between two groups was performed using a two-tailed unpaired student's t-test. One-way ANOVA was used to compare three or more groups, followed by Bonferroni correction for multiple comparisons. All analyses were performed using SPSS 22.0. A P value of <0.05 is considered significant.

## Supplementary Material

Supplementary figures.

## Abbreviations

AKI: Acute kidney injury
TECs: Tubular epithelial cells
cAKI: cisplatin-induced AKI
TRF1: Telomeric repeat binding factor 1
CDK12: Cyclin-dependent kinase 12
DDR: DNA damage response
WT: Wild-type
PAS: Periodic acid-Schiff
HE: Hematoxylin-eosin
TUNEL: Terminal deoxynucleotidyl transferase mediated dUTP nick end labeling
Q-FISH: Quantitative telomere fluorescence *in situ* hybridization
TIFs: Telomeric dysfunction-induced foci
MTSs: Multiple telomeric signals
NC: Negative control
I/R: Ischemia/reperfusion
SCr: Serum creatinine
BUN: Blood urea nitrogen
FBS: Fetal bovine serum
SD: Standard deviation
RT-PCR: real-time PCR
