# Supplementary material for: MiR-155 deficiency protects renal tubular epithelial cells from telomeric and genomic DNA damage in cisplatin-induced acute kidney injury
Source: Theranostics. 2022 Jun 6;12(10):4753–66. doi: 10.7150/thno.72456 (PMC9254246; doi:10.7150/thno.72456)

Figure S1

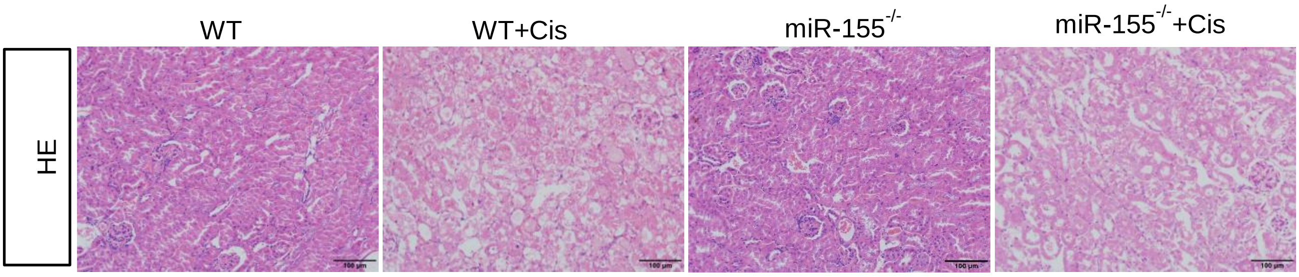

Figure S1. Representative kidney histology as shown by HE staining 72 h after saline or cisplatin injection.

Figure S2

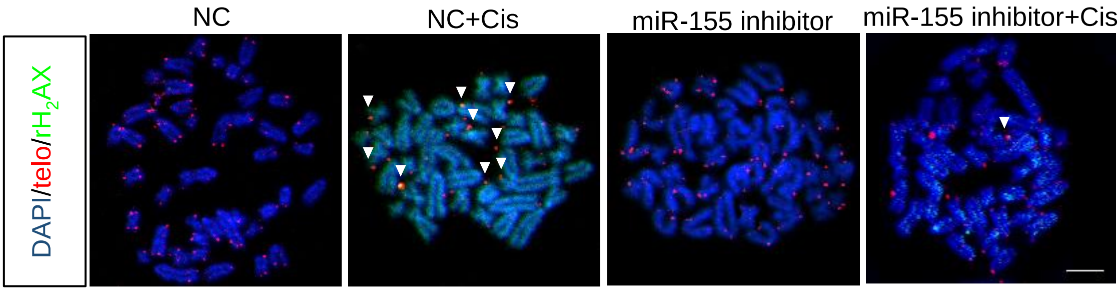

Figure S2. Telomere DNA damage in TECs treated with cisplatin, as demonstrated by the co-localization of γH2AX (green) and telomeres (red) in the immunofluorescence. White arrows indicate TIFs. Scale bars: 10 μm. TIFs, telomere dysfunction induced foci.

Figure S3

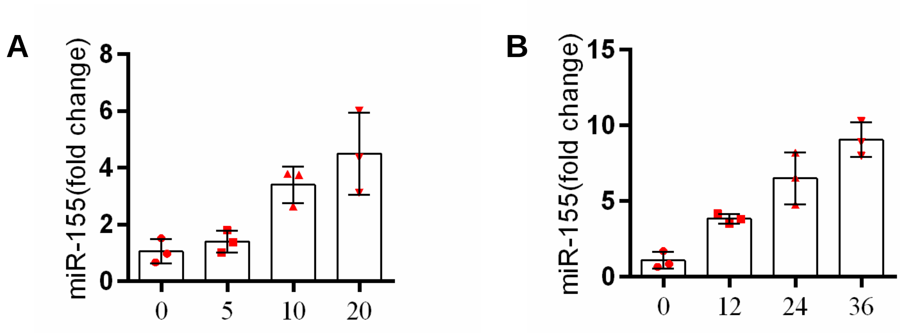

Figure S3. (A) qPCR of miR-155 in HK-2 cells (n = 3). HK-2 cells were treated with cisplatin 0, 5, 10, 20 μM for 24 h. (B) qPCR of miR-155 in HK-2 cells (n = 3). HK-2 cells were treated with cisplatin 20 μM for 0, 12, 24, 36 h.

Figure S4

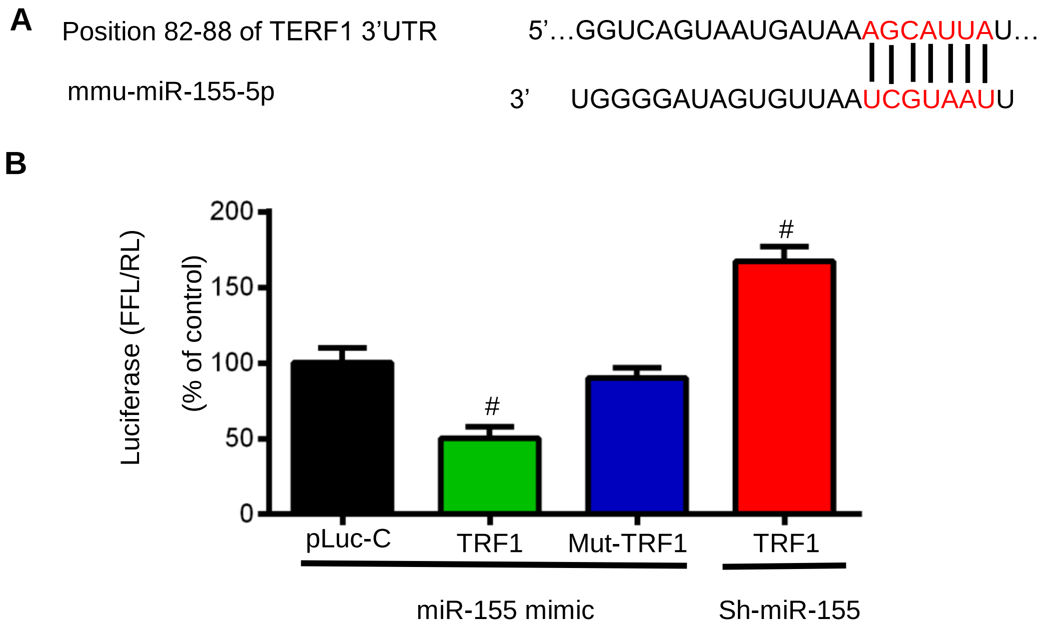

Figure S4. mmu-miR-155 targets TRF1. (A) The schematic diagram depicted the predicted binding site of mmu-miR-155 targeting the 3'-UTR of TRF1. (B) Luciferase reporter assay determined TRF1 as a bona fide target of miR-155 (n = 3). pLuc-ctrl (pMIR-REPORT luciferase); Mut, mutant; The data represent the mean ± SD; # P < 0.05 versus mimic control.

Original data 1.

Unedited images for figure 2C

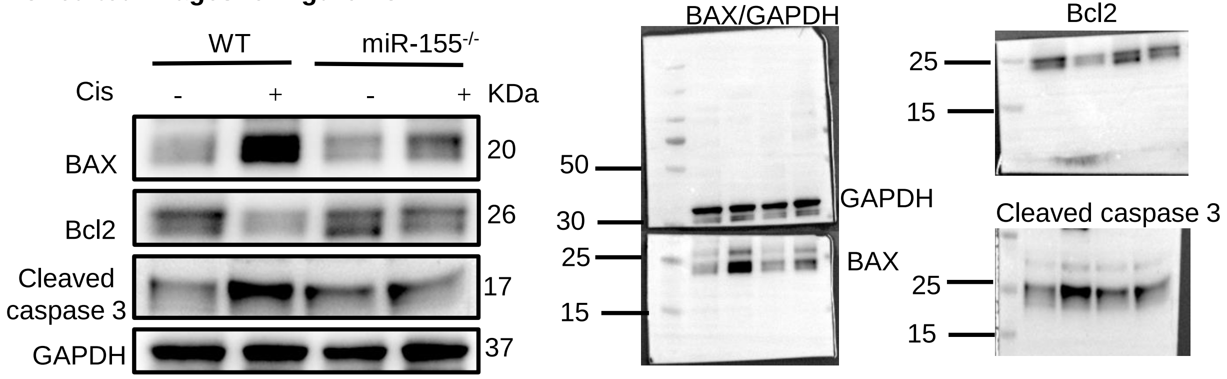

Unedited images for figure 2I

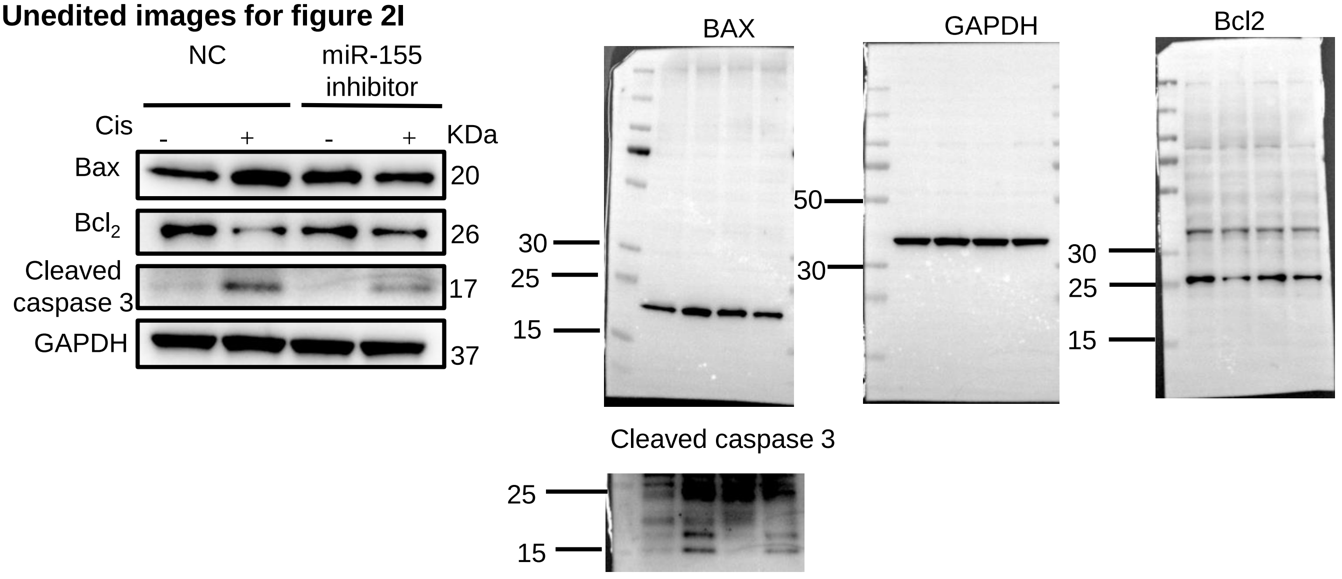

Original data 2.

Unedited images for figure 3C

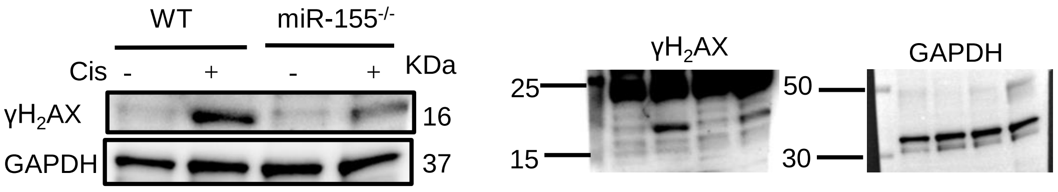

Unedited images for figure 3G

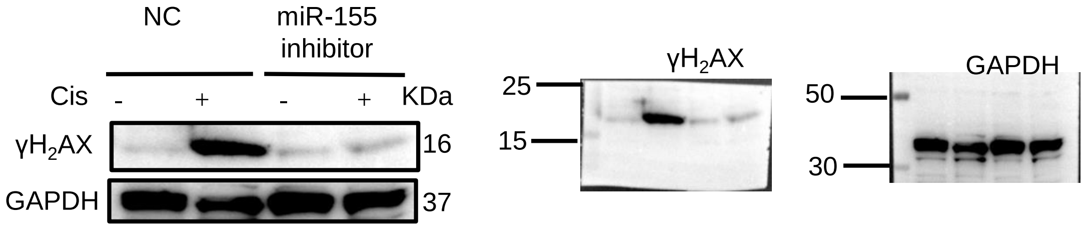

Original data 3.

Unedited images for figure 5C

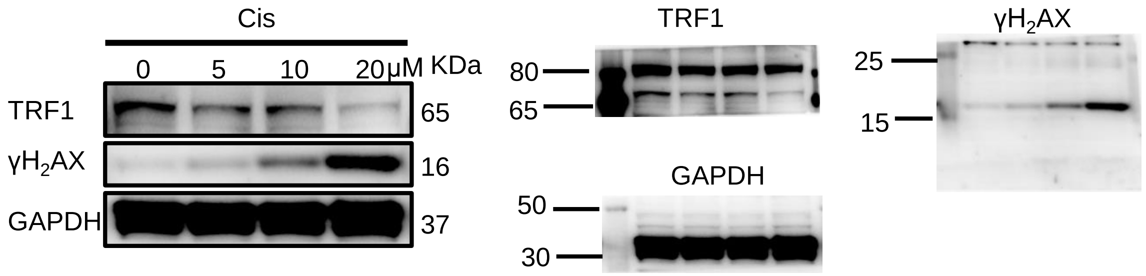

Unedited images for figure 5F

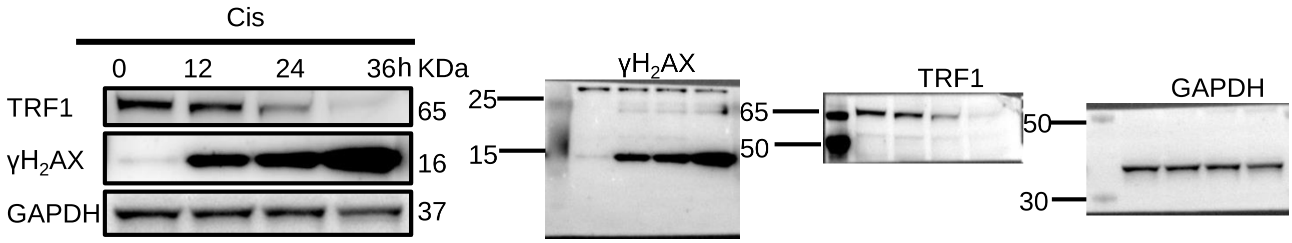

Unedited images for figure 5K

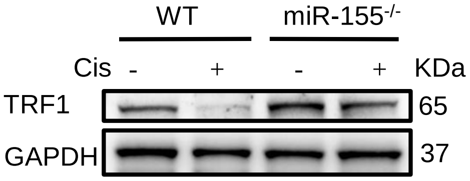

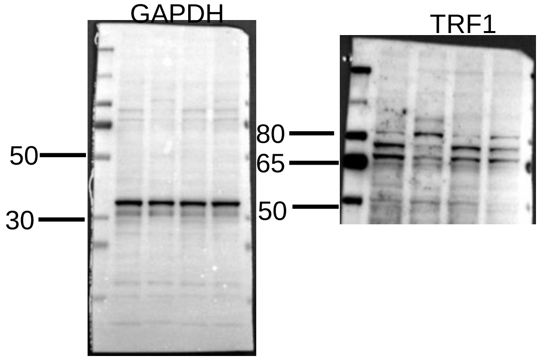

Unedited images for figure 5M

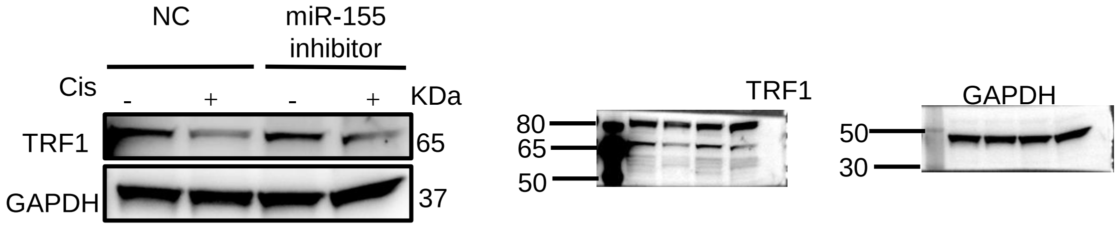

Unedited images for figure 5R

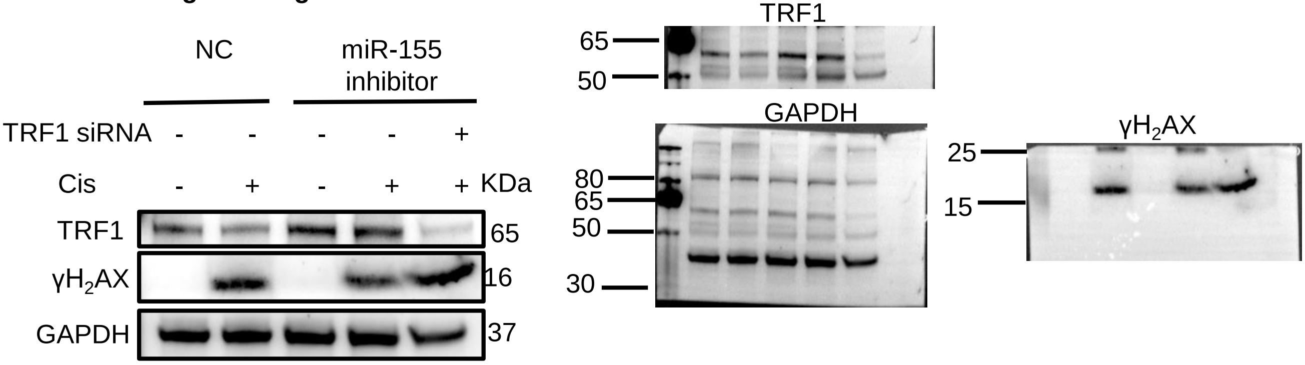

Original data 4.

Unedited images for figure 6C

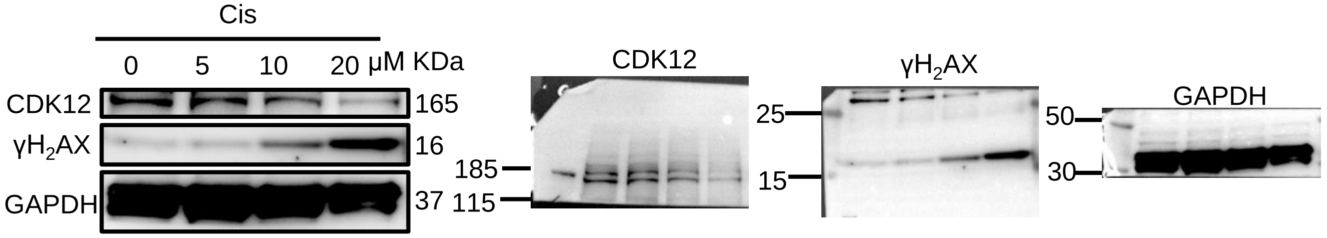

Unedited images for figure 6F

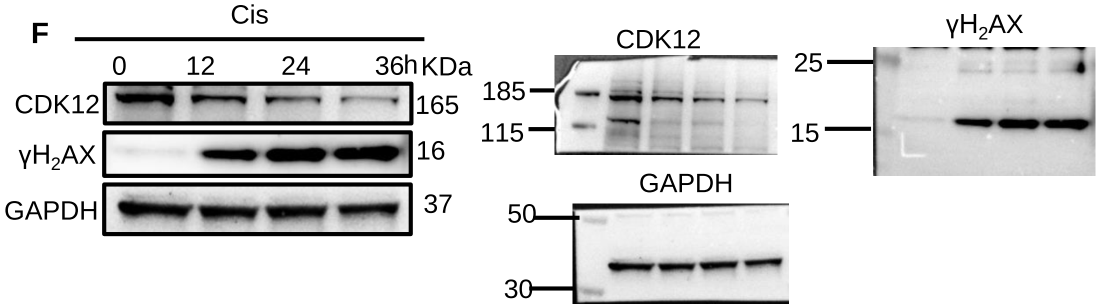

Unedited images for figure 6I

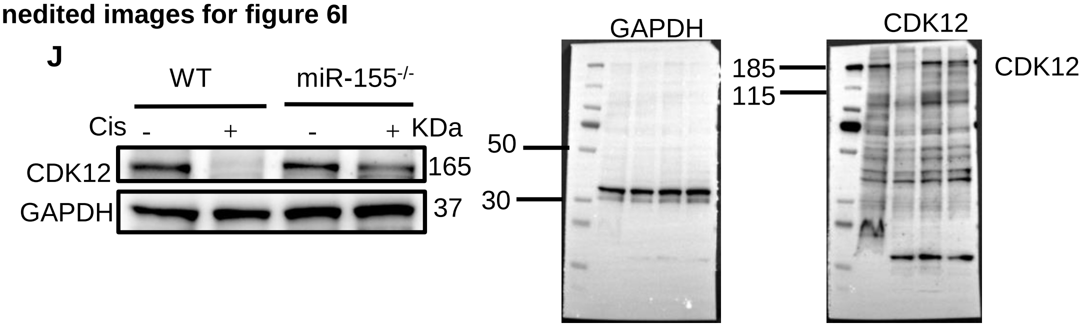

Unedited images for figure 6M

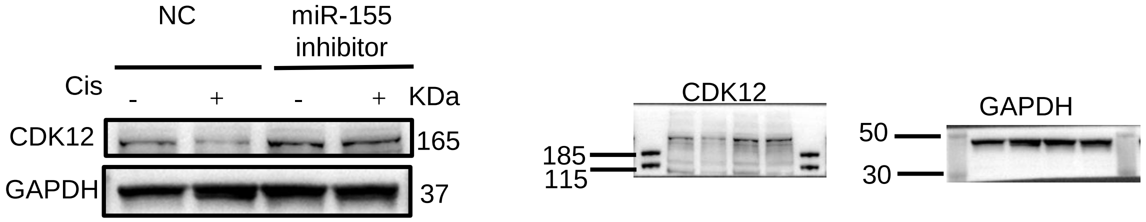

Unedited images for figure 6Q

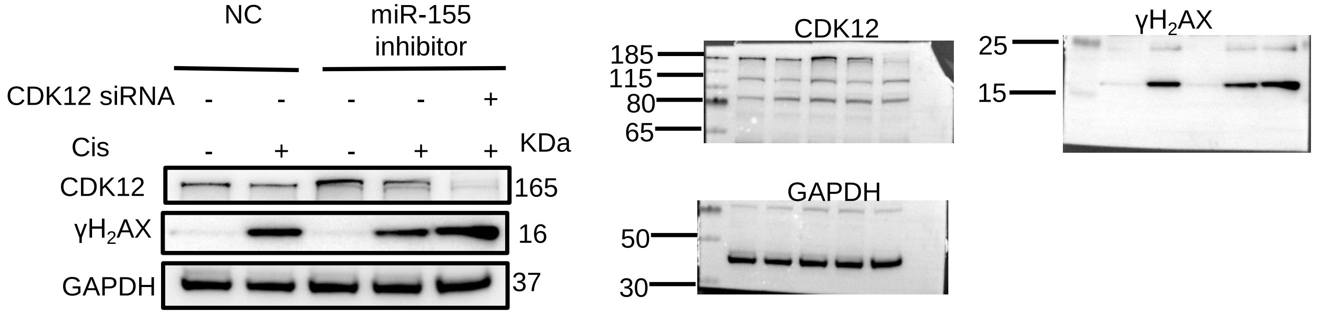

Supplement: Supplementary file 1 — Supplementary figures. [file thnov12p4753s1.pdf]
